# Supplementary material for: Integrative Evaluation of the Clinical Significance Underlying Protein Arginine Methyltransferases in Hepatocellular Carcinoma
Source: Cancers (Basel). 2023 Aug 20;15(16):4183. doi: 10.3390/cancers15164183 (PMC10453297; doi:10.3390/cancers15164183)
Supplement: Supplementary file 1 [file cancers-15-04183-s001.zip › Supplement Figure S2.pdf]

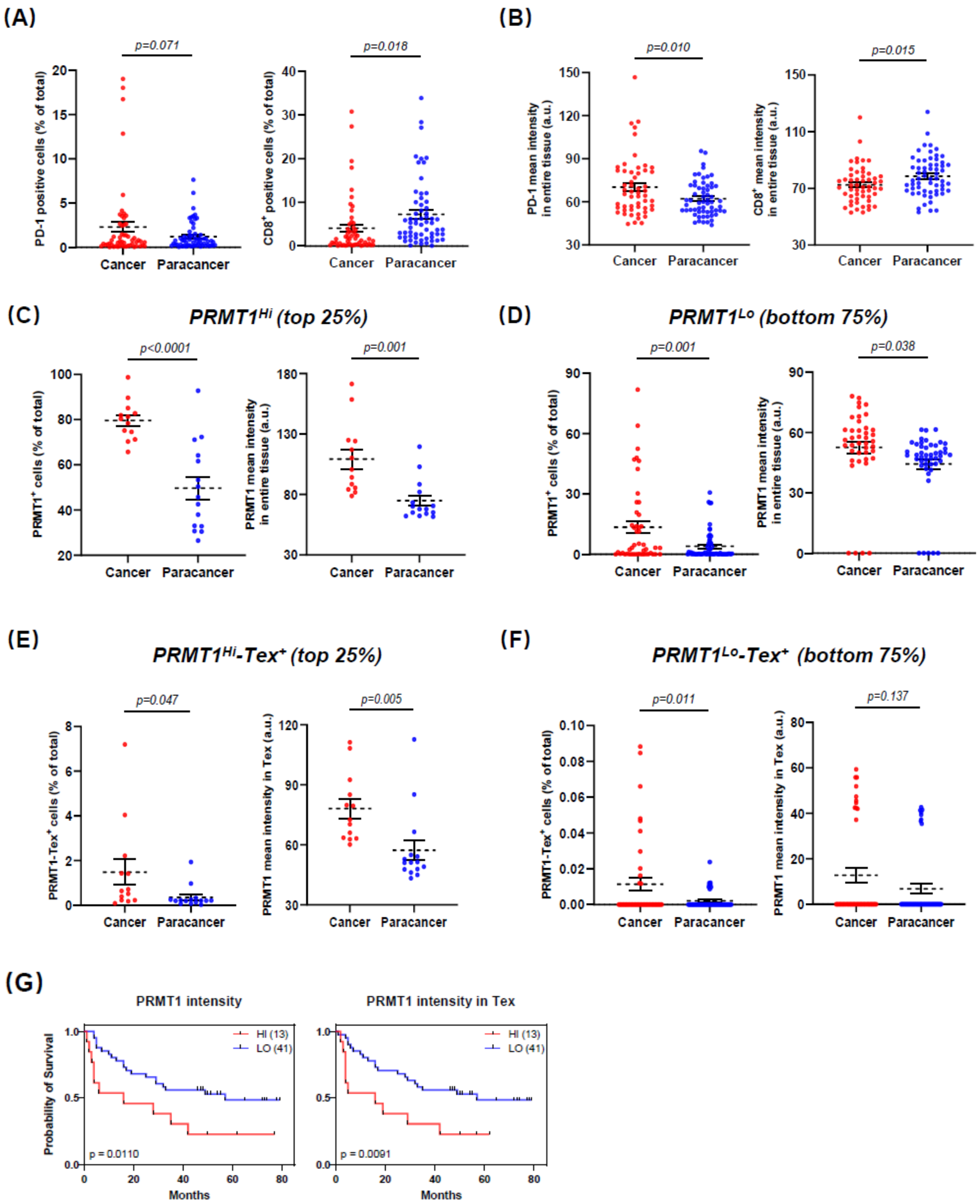

Supplement Figure S2. Prognostic significance of PRMT1 and PRMT1-Tex in HCC patients. (A, B) The expression patterns (positive rate and expression intensity) of PD-1 are statistically higher in HCC, compared to normal tissues, contrary to those of CD8+. (C-F) The expression patterns of PRMT1 are statistically higher in HCC with both PRMT1Hi (C) and PRMT1Lo (D), consistent with the expression patterns of PRMT1 in Tex of HCC (E,F), compared to normal tissues. (G) Survival probabilities (Kaplan–Meier curves) show the correlation between PRMT1 (or PRMT1-Tex) expression intensity and overall survival. The R package, multipleROC, divided into two groups (i.e., Hi vs Lo) according to the optimal gene expression level of PRMT1 (or PRMT1-Tex). The R packages, survminer and survival, estimated the survival probability.
