# Supplementary material for: Integrative Evaluation of the Clinical Significance Underlying Protein Arginine Methyltransferases in Hepatocellular Carcinoma
Source: Cancers (Basel). 2023 Aug 20;15(16):4183. doi: 10.3390/cancers15164183 (PMC10453297; doi:10.3390/cancers15164183)
Supplement: Supplementary file 1 [file cancers-15-04183-s001.zip › Supplement Table 1, 2.pdf]

Supplement Table S1. Clinical characteristics of the patient population with PRMT1 expression

	variables	PRMT1⁺		Total	*p* value
		high	low		
Age (year)					0.7657
	≤60	16	27	43	
	>60	4	7	11	
Grade					0.8152
	I/II	10	23	33	
	III	7	14	21	
Tumor size					0.7818
	≤3.5cm	4	10	14	
	>3.5cm	15	25	40	
TNM stage					0.2017
	I/II	7	18	25	
	III/IV	13	16	29	

Supplement Table S2. Clinical characteristics of the patient population with PRMT1 expressed in Tex

	variables	PRMT1-Tex⁺		Total	*p* value
		high	low		
Age (year)					0.7625
	≤60	7	36	43	
	>60	2	9	11	
Grade					0.7600
	I/II	4	29	33	
	III	4	17	21	
Tumor size					0.9410
	≤3.5cm	3	11	14	
	>3.5cm	7	33	40	
TNM stage					0.9274
	I/II	5	20	25	
	III/IV	5	24	29	
