# Supplementary material for: Integrative Evaluation of the Clinical Significance Underlying Protein Arginine Methyltransferases in Hepatocellular Carcinoma
Source: Cancers (Basel). 2023 Aug 20;15(16):4183. doi: 10.3390/cancers15164183 (PMC10453297; doi:10.3390/cancers15164183)
Supplement: Supplementary file 1 [file cancers-15-04183-s001.zip › supplementary Figure S1.pdf]

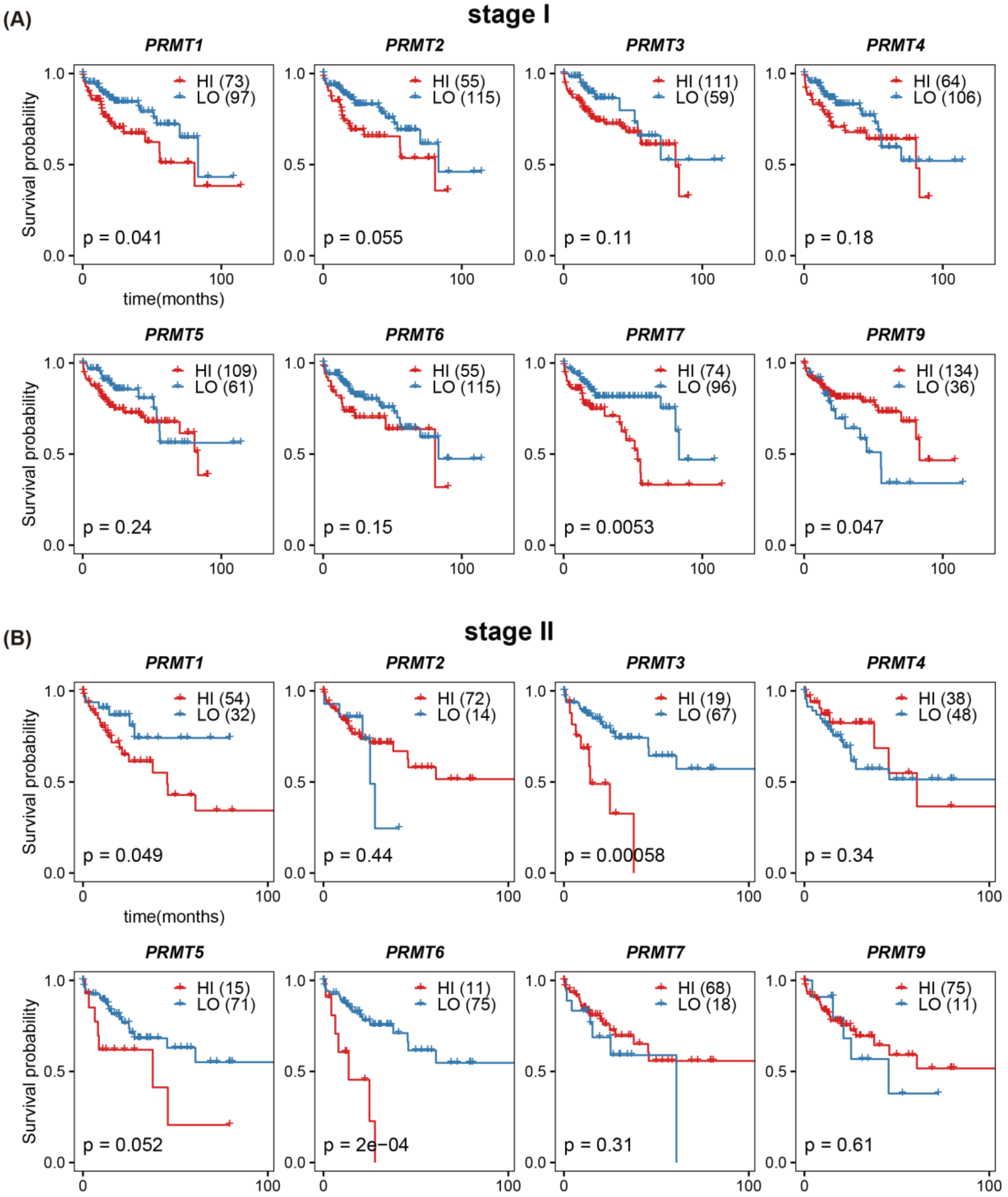


**Supplementary Figure S1. Prognostic significance of PRMTs in patients with different stages of HCC. (A, B)** Survival probabilities (Kaplan–Meier curves) in different stages (Stage I and II) of HCC show the association between hepatic PRMT expression and overall survival. The patients were divided into two groups based on their PRMT gene expression levels: one with high expression and the other with low expression, determined by the R package, *multipleROC*. Survival probabilities were estimated using the R packages, *survminer* and *survival*.
